# Poly (ADP-ribose) Interacts With Phosphorylated α-Synuclein in Post Mortem PD Samples

**DOI:** 10.3389/fnagi.2021.704041

**Published:** 2021-06-18

**Authors:** Laura N. Puentes, Zsofia Lengyel-Zhand, Ji Youn Lee, Chia-Ju Hsieh, Mark E. Schneider, Kimberly J. Edwards, Kelvin C. Luk, Virginia M.-Y. Lee, John Q. Trojanowski, Robert H. Mach

**Affiliations:** ^1^Department of Systems Pharmacology and Translational Therapeutics, Perelman School of Medicine, University of Pennsylvania, Philadelphia, PA, United States; ^2^Department of Radiology, Perelman School of Medicine, University of Pennsylvania, Philadelphia, PA, United States; ^3^Department of Pathology and Laboratory Medicine, Institute on Aging and Center for Neurodegenerative Disease Research, Perelman School of Medicine, University of Pennsylvania, Philadelphia, PA, United States

**Keywords:** poly (ADP-ribose), PARP-1, alpha-synclein, Parkinson’s disease (PD), neurodegeneration

## Abstract

Poly (ADP-ribose) (PAR) is a negatively charged polymer that is biosynthesized by Poly (ADP-ribose) Polymerase-1 (PARP-1) and regulates various cellular processes. Alpha-synuclein (αSyn) is an intrinsically disordered protein (IDP) that has been directly implicated with driving the onset and progression of Parkinson’s disease (PD). The mechanisms by which α-synuclein (αSyn) elicits its neurotoxic effects remain unclear, though it is well established that the main components of Lewy bodies (LBs) and Lewy neurites (LNs) in PD patients are aggregated hyperphosphorylated (S129) forms of αSyn (pαSyn). In the present study, we used immunofluorescence-based assays to explore if PARP-1 enzymatic product (PAR) promotes the aberrant cytoplasmic accumulation of pαSyn. We also performed quantitative measurements using *in situ* proximity ligation assays (PLA) on a transgenic murine model of α-synucleinopathy (M83-SNCA^∗^A53T) and post mortem PD/PDD patient samples to characterize PAR–pαSyn interactions. Additionally, we used bioinformatic approaches and site-directed mutagenesis to identify PAR-binding regions on αSyn. In summary, our studies show that PAR–pαSyn interactions are predominantly observed in PD-relevant transgenic murine models of αSyn pathology and post mortem PD/PDD patient samples. Moreover, we confirm that the interactions between PAR and αSyn involve electrostatic forces between negatively charged PAR and lysine residues on the N-terminal region of αSyn.

## Introduction

A characteristic feature in the pathogenesis of Parkinson’s disease (PD) involves the accumulation of alpha-synuclein (αSyn) protein within the cytoplasm of brain cells (Maries et al., 2003; Waxman and Giasson, 2009) — an event that underlies the molecular basis of PD pathology (Bridi and Hirth, 2018; Zeng et al., 2018). While the exact mechanisms associated with PD progression are unknown, it is well understood that the intracellular aggregation of αSyn is directly linked to the neurodegeneration found in PD (Maries et al., 2003). αSyn is a protein that primarily exists as a natively unfolded soluble monomer (Lashuel et al., 2013). In neurons, αSyn is believed to function in a variety of synaptic processes, including vesicle trafficking and recycling (Kahle et al., 2000; Murphy et al., 2000; Cabin et al., 2002). Depending on the environment, αSyn can undergo a variety of dynamic conformational changes, which include the formation of α-helix-rich tetramers (Dettmer et al., 2015), partially folded α-helical forms (due to interactions with biological membranes), transitioning into oligomeric species, and producing toxic fibrils that are insoluble and resistant to protease activity (Vermaas and Tajkhorshid, 2014). The resulting effect of the latter is a loss in the original protein function and damage in the affected neurons (Perez and Hastings, 2004). In PD, αSyn accumulates into higher-order aggregates known as Lewy bodies (LBs) and Lewy neurites (LNs) (Spillantini et al., 1997).

In the last decade, extensive research has been done exploring the role of nuclear protein Poly (ADP-ribose) Polymerase-1 (PARP-1) in promoting neurodegeneration (Outeiro et al., 2007; Yunjong et al., 2014). Studies have shown that PARP-1 hyperactivation depletes NAD^+^, induces an accumulation of Poly (ADP-ribose) (PAR), and triggers mitochondrial damage in Alzheimer’s disease (AD) (Martire et al., 2015), Huntington’s disease (HD) (Cardinale et al., 2015), amyotrophic lateral sclerosis (ALS) (Kim et al., 2004), ischemic brains (Moroni, 2008), and PD (Yunjong et al., 2014). PAR is primarily synthesized by PARP-1 from NAD^+^ in the nucleus of cells (Luo and Kraus, 2012); it regulates cellular processes such as modulating protein localization through covalent (aspartic, glutamic, or lysine residues) and non-covalent interactions via PAR-binding motifs (PBMs) on target proteins (Pleschke et al., 2000). Several lines of evidence show that increased levels of intracellular PAR promote liquid demixing and irreversible aggregation of intrinsically disordered proteins (IDPs) (Altmeyer et al., 2015). Moreover, PAR and PARylated proteins have been shown to interact directly with pathogenic protein states, such as, Aβ (Martire et al., 2013), TDP43 (McGurk et al., 2018), and hnRNP-A1 (Duan et al., 2019). Thereby, affecting the aggregation kinetics of these proteins, potentiating toxicity, and promoting cell-to-cell transmission. As such, it has been suggested that the association of PAR and protein aggregates may serve as a feed-forward mechanism that amplifies neurotoxicity and drives neurodegeneration (Narne et al., 2017). A seminal study by Kam et al. (2018), revealed that αSyn preformed fibrils (PFF) increase intracellular oxidant levels which result in DNA damage and activation of PARP-1, leading to the intraneuronal accumulation of PAR and cell death via Parthanatos (Kam et al., 2018). It was also reported that PAR binds αSyn PFF resulting in a more stable PFF that displays faster fibrillization kinetics and higher neurotoxicity.

In the present study, we employed the use of a human neuroblastoma line overexpressing wild type αSyn (SH-SY5Y-αSyn) to gather physiologically relevant information on the role of PAR in promoting the accumulation of phosphorylated αSyn (pαSyn). We also performed *in situ* proximity ligation assays (PLA) to gain respective insight into the pathophysiological significance of PAR–pαSyn interactions, and utilized site-directed mutagenesis, immunodot blots, and molecular docking studies to elucidate the nature of these interactions. Altogether, our results support the notion that PAR plays a role in the aggregation pathway of αSyn and reinforce the importance of investigating small-molecule inhibitors of PARP-1 as disease modifying therapies for PD.

## Results

### PAR Colocalizes With Phosphorylated (S129) αSyn Aggregates *in vitro*

In physiological settings, approximately 4% of soluble αSyn is phosphorylated at amino acid residue S129 (pαSyn) (Anderson et al., 2006; Tenreiro et al., 2014). Correlations have been established between pαSyn status and pathological conditions (Oueslati, 2016; Wang et al., 2012). In LBs, it is estimated that up to 90% of αSyn is phosphorylated at S129 (Anderson et al., 2006). Furthermore, pαSyn is observed in other synucleinopathies (neurodegenerative diseases characterized by abnormal accumulation of αSyn aggregates) as well, including dementia with LBs (DLB) (Kim et al., 2014) and multiple system atrophy (MSA) (Wakabayashi et al., 1998). In addition, increased levels of pαSyn have also been reported in PD-like transgenic murine models (Neumann et al., 2002).

**FIGURE 1:**
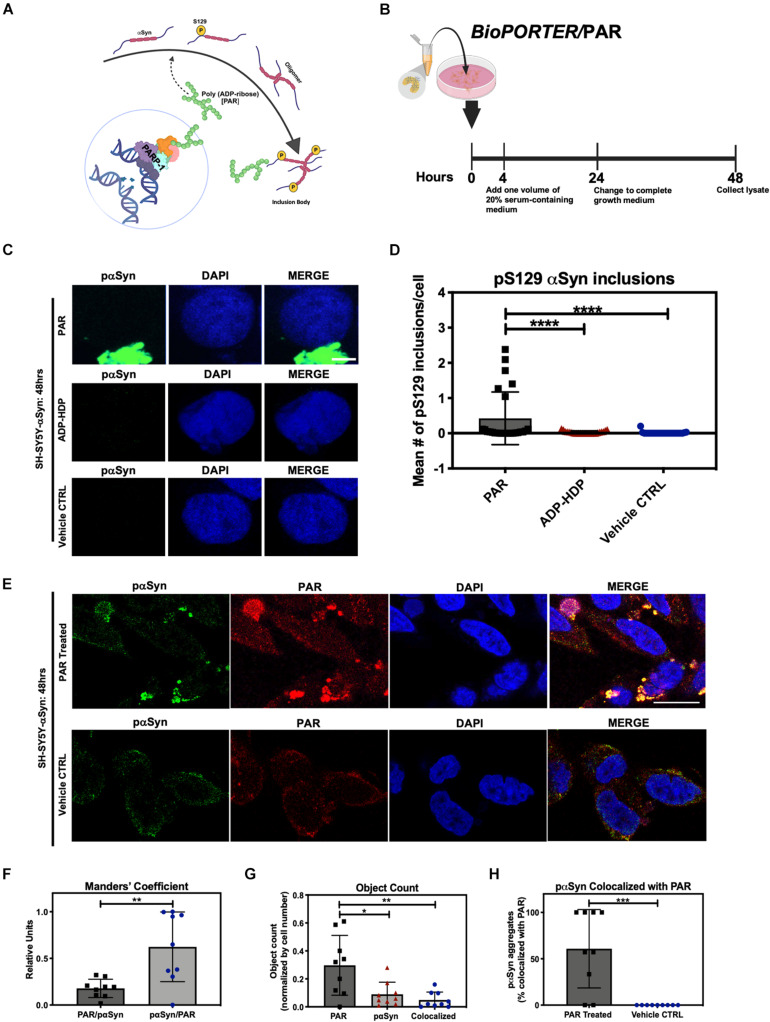
Poly (ADP-ribose) (PAR) colocalizes with pαSyn. **(A)** Proposed mechanism of PAR-induced pαSyn accumulation, whereby PARP-1 hyperactivation due to DNA damage results in excess PAR production, leading to cytoplasmic pαSyn accumulation and pathogenic PAR–pαSyn interactions. **(B)** Experimental scheme of *BioPORTER*-mediated transfection of PAR polymer into SH-SY5Y-αSyn neuroblastoma cells. **(C)** Representative immunostain of pαSyn (green) and DAPI (blue) in SH-SY5Y-αSyn cells 48 h post treatment with either 50 nM PAR or ADP-HDP vs. *BioPORTER* alone (vehicle control). Scale bar 5 μm. **(D)** Quantification of pαSyn inclusions (aggregates larger than 1 μm) in PAR treated, ADP-HDP treated, and *BioPORTER* alone (vehicle control) samples. Bars represent mean ± SD. Two-way ANOVA followed by Tukey’s *post hoc* test (*n* = 3). ****P < 0.0001. **(E)** Representative IF immunostain of pαSyn (green), PAR polymer (red), and DAPI (blue) in SH-SY5Y-αSyn cells 48 h post treatment with 50 nM PAR vs. *BioPORTER* alone (vehicle control). Scale bar 10 μm. **(F)** Manders’ overlap coefficient analysis between total PAR over pαSyn inclusions (PAR/pαSyn) and pαSyn inclusions over total PAR (pαSyn/PAR) in the PAR treated samples, whereby an overlap coefficient of 0.5 implies that 50% of both objects (i.e., pixels) overlap. Bars represent mean ± SD. Student’s two-tailed *t*-test (*n* = 3). **P < 0.002. **(G)** Average object count in the PAR treated samples for the following objects: PAR, pαSyn inclusions, and colocalized PAR-pαSyn inclusions. Object counts were normalized by DAPI signal (i.e., cell number). Bars represent mean ± SD. Two-way ANOVA followed by Tukey’s *post hoc* test (*n* = 3). *P < 0.02, **P < 0.0045. **(H)** Colocalization analysis comparing the number of pαSyn inclusions colocalized with PAR immunostain in PAR treated and *BioPORTER* alone (vehicle control) samples. Images were captured using Zeiss LSM 710 confocal (40×/1.4 Oil) microscope. Bars represent mean ± SD. Student’s two-tailed *t*-test (*n* = 3). ***P < 0.0005. Graphical symbols represent *fields-of-view* containing 50–70 cells each.

The processes by which native αSyn transitions from a monomeric state to a pathogenic aggregate form are unknown. As such, identifying the underlying factors that drive abnormal αSyn assembly are vital to understand the pathogenesis of PD. In this study, we asked whether the addition of exogenous PAR could promote the cytoplasmic accumulation of pαSyn *in vitro* (Figure 1A). To address this question, we employed the use of a protein transfection system, *BioPORTER*, to deliver a physiologically relevant dose of PAR polymer (50 nM) into SH-SY5Y-αSyn cells (Figure 1B). The rationale for the use of *BioPORTER –* instead of a genotoxic agent like MNNG (Lábaj et al., 2003; Carrozza et al., 2009; Gassman et al., 2012) – was to develop a neuronal-like cell model that recapitulated the effects of PARP-1 hyperactivation (i.e., elevated PAR) in a genomically stable setting. Additionally – and in parallel – we also used *BioPORTER* to deliver 50 nM of adenosine diphosphate (hydroxymethyl)pyrrolidinediol (ADP-HDP) (Slama et al., 1995) into SH-SY5Y-αSyn cells to assess if the stable NH-analog of ADP-ribose was sufficient to induce intracellular αSyn aggregation. After a 48 h incubation, the cells were immunostained with an antibody directed toward pαSyn. Using fluorescence microscopy, we identified pαSyn inclusions (∼1 μm length) in the cytoplasm of PAR treated cells (Figures 1C,D). We also noted that PAR signal overlapped with ∼ 60% of these cellular pαSyn inclusions when co-immunostained with a PAR-specific antibody (Figures 1E,F).

Our studies showed that the addition of exogenous PAR led to the accumulation of cytosolic pαSyn inclusions (Figures 1C,D) by 48 h. By contrast, these pαSyn inclusions were not observed in the ADP-HDP treated or vehicle control samples (Figures 1C,D). In addition, quantification of co-immunostained samples using image processing software, indicated that while over half of the pαSyn inclusions were colocalized with PAR signal (Figures 1G,H), the majority of PAR signal was not colocalized with pαSyn (Figure 1F). The latter was not surprising given the diverse roles that PAR plays in the cell (David et al., 2009; Leung, 2014).

### PAR Interacts With Phosphorylated (S129) αSyn *in vitro*

To directly measure the interactions between PAR and pαSyn in our cell model, we employed *in situ* PLA (Alam, 2018), which allowed us to record the prevalence of PAR–pαSyn interactions with greater sensitivity and specificity when compared to traditional immunoassays.

**FIGURE 2:**
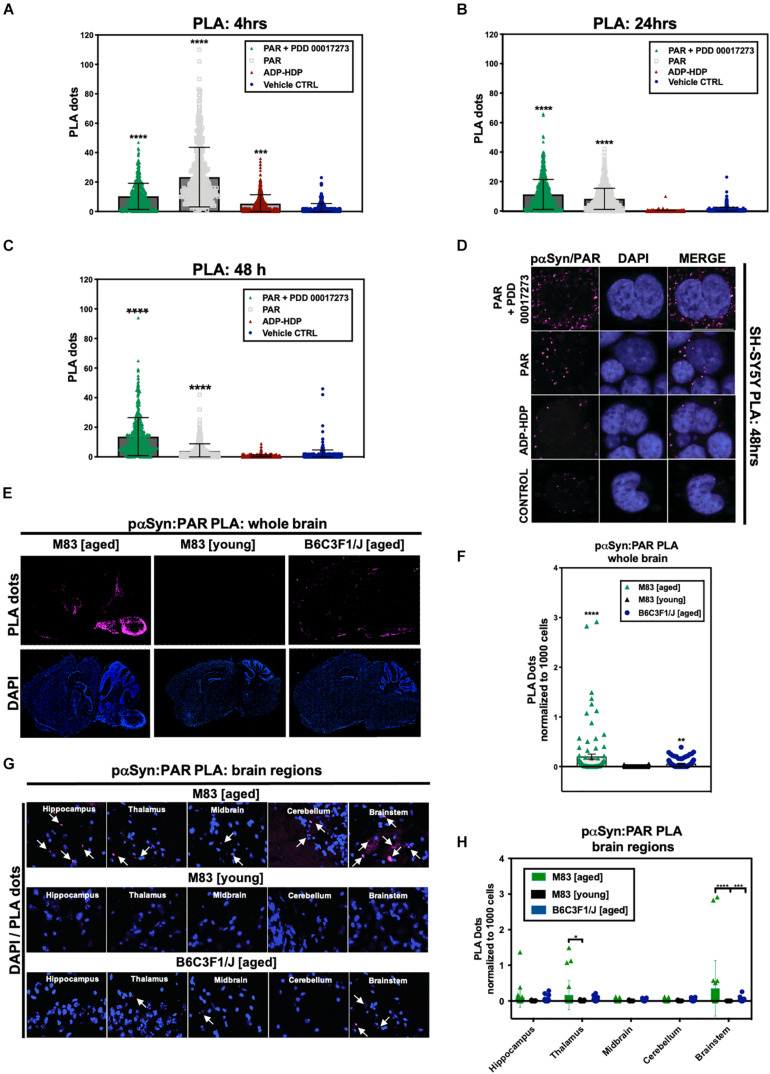
Poly (ADP-ribose) interacts with phosphorylated (S129) αSyn in pathological settings. Quantification from proximity ligation assays (PLA) measuring pαSyn and PAR interactions in SH-SY5Y-αSyn cells at **(A)** 4 h, **(B)** 24 h, and **(C)** 48 h post treatment with either PAR, PAR + 1 μM PDD00017273 (PARGi) or ADP-HDP vs. *BioPORTER* alone (vehicle control). Bars represent means ± SD. One-way ANOVA followed by Tukey’s *post hoc* test (*n* = 3). ***P < 0.0004, ****P < 0.0001 **(D)** Representative DAPI and PLA ROI images showing PLA dots (pink); these dots indicate direct interactions between pαSyn and PAR in PAR treated, ADP-HDP treated and *BioPORTER* alone (vehicle control) samples at 48 h. Scale bar 10 μm. **(E)** Representative DAPI (bottom panel) and PLA (top panel) whole brain images from M83 Tg aged, M83 Tg young, and B6C3F1/J aged mice. **(F)** Quantification of whole brain PLA levels in M83 Tg aged, M83 Tg young and B6C3F1/J mice (*n* = 3 per group). Each bar represents means ± SD. One-way ANOVA. **P < 0.002, ****P < 0.0001. **(G)** Representative PLA staining (white arrows) of ROIs obtained from 20× merge images. **(H)** Quantification of different brain regions in M83 Tg aged, M83 Tg young, and B6C3F1/J aged mice. Images were captured using Zeiss Axio Widefield (20×/0.8) microscope. Bars represent means ± SD. Two-way ANOVA followed by Tukey’s *post hoc* test (*n* = 3). *P < 0.025, ***P < 0.0002, ****P < 0.0001.

In addition to PAR/*BioPORTER* delivery, we also added a small molecule PARG inhibitor (PDD 00017273) (James et al., 2016) to limit the degradation of the exogenous PAR. PARG is an enzyme that regulates intracellular PAR levels via its exo- and endoglycosidase activities (Le May et al., 2012). Thus, to reduce PAR catabolism, we pre-treated SH-SY5Y-αSyn cells with 1 μM PARG inhibitor PDD 00017273, 1 h prior to *BioPORTER* delivery of 50 nM PAR. Results from the PLA showed an increase in signal for both the PAR and PAR + PARGi treated samples when compared to ADP-HDP treated and vehicle control samples at 4 h (Figure 2A), 24 h (Figure 2B), and 48 h (Figures 2C,D). Notably, PLA signal for the PAR + PARGi treatment condition remained constant for all three time points (4, 24, and 48 h), while PLA signal for the PAR-only condition decreased from ∼18 PLA dots/cell at 4 h to ∼12 PLA dots/cell at 24 h to ∼5 PLA dots/cell at 48 h. Results from a PAR ELISA confirmed that the decrease in PLA signal for the PAR-only condition was likely due to a decrease in PAR levels from 4 to 48 h; this decrease was presumably due to the degradation of PAR by PARG and ADP-ribosylhydrolase 3 (ARH3) (Mashimo et al., 2013) (Supplementary Figure 1A). Interestingly, we also recorded an increase in PLA signal for ADP-HDP treated samples at 4 h (Figure 1A). Additional studies revealed that this increase in signal was likely due to elevated PAR levels at 4 h resulting from ADP-HDP-mediated PARG inhibition (Supplementary Figure 1B). After 24 h, the PLA signal for the ADP-HDP treated samples returned to baseline levels (Figure 2B), this decrease was likely due to the rapid degradation of ADP-HDP by phosphodiesterases in the cell (Wang et al., 2014).

Altogether, the evidence from our cell model suggests that the exogenous addition of PAR promotes the formation of hyperphosphorylated αSyn inclusions in the cytoplasm of SH-SY5Y-αSyn cells and that PAR likely stabilizes pαSyn inclusions, as evidenced by our PAR–pαSyn colocalization and PLA studies.

### PAR and pαSyn Interactions Are Prevalent in a PD-Like Transgenic Mouse Model of α-Synucleinopathy

In order to assess if PAR–pαSyn interactions are present in αSyn pathology, we used a transgenic (Tg) murine model of α-synucleinopathy (M83 SCNA^∗^A53T) that develops a PD-like phenotype with age (Bétemps et al., 2014). The Tg murine line (M83) used in this study overexpresses a form of human αSyn that harbors a point mutation at amino acid residue 53 (A53T); this point mutation has been directly implicated in familial early onset PD (Li et al., 2002). Information on the animals used in this study can be found in Supplementary Table 2.

The Tg mice were separated into two groups: M83 Tg young (less than 12 months) and M83 Tg aged (12 months or older). To control for age-related effects, we also used a non-transgenic murine line (B6C3F1/J) in our studies. Following euthanasia, murine brains were dissected and hemisected in the sagittal plane. Immunostaining for endogenous PAR was carried out to assess PARP-1 activity. Our studies revealed that PAR signal intensity increased with age in both M83 Tg and non-Tg mice (Supplementary Figure 2A), an increase that was particularly pronounced in the cerebral cortex, hippocampus, and cerebellum. We also observed a notable increase in signal in the cortex, cerebellum, thalamus, and brain stem regions of an M83 Tg aged (17 months) mouse compared to an age-matched control. Likewise, a study by Mao et al. (2020) recently showed that both PAR and PARP-1 are elevated in SNpc and cortical brain regions of M83 Tg mice compared to WT controls.

In addition to PAR, we also immunostained for pαSyn to assess whole brain expression in both M83 Tg and non-Tg mice (Supplementary Figure 2B). Notably, we observed a remarkable increase in pαSyn expression in an M83 Tg aged (17 months) sample – with maximum signal output measured in the brainstem, midbrain, thalamus, hypothalamus, and cerebral cortex regions (Supplementary Figure 2B).

To measure the prevalence of PAR–pαSyn interactions, we performed *in situ* PLA on frozen brain sections from all three animal groups: M83 Tg young, M83 Tg aged and non-Tg (B6C3F1/J). To limit non-specific PLA signal, adjacent brain tissue sections were incubated without primary antibody, subsequent imaging parameters (i.e., exposure time and depth of field) were then adjusted in order to acquire detectable signal above background. To ensure consistency between experimental models, we used the same primary antibodies (anti-PAR and anti-pαSyn, Supplementary Table 1) for our cell and animal brain tissue PLA. Results from our studies revealed that PLA signal for PAR–pαSyn was strongest in M83 Tg vs. non-Tg mice (Figures 2E,F). Analogously, M83 Tg aged vs. M83 Tg young mice differed significantly in PLA signal (Figures 2E,F) – with the strongest signal differential detected in the brainstem region of the M83 Tg aged group (Figures 2G,H). Similarly, increased PLA signal was also observed in an M83 Tg aged mouse (18 months) when using primary antibodies against PAR and total αSyn (anti-PAN-αSyn) (Supplementary Figure 2C), thus confirming that PAR interacts with both phosphorylated and non-phosphorylated αSyn. In addition, we noted that M83 Tg brain tissue samples with the highest pαSyn expression also had the greatest PLA signal output, suggesting that pαSyn-PAR PLA signal may be directly tied to the amount of pathology (i.e., pαSyn expression) present in a given sample.

Overall, our studies revealed that PLA signal was highest in anatomical brain regions most commonly associated with αSyn pathology in the M83 Tg aged group (Leung, 2014) (Figures 2E,F); these findings are in accordance with our observations from the SH-SY5Y-αSyn cell model, which show that PAR–pαSyn interactions are prevalent in pathogenic states involving both αSyn aggregation and elevated PAR levels.

### PAR–pαSyn Interactions Are Observed in Post Mortem Brain Tissue From PD/PDD Patients

**FIGURE 3:**
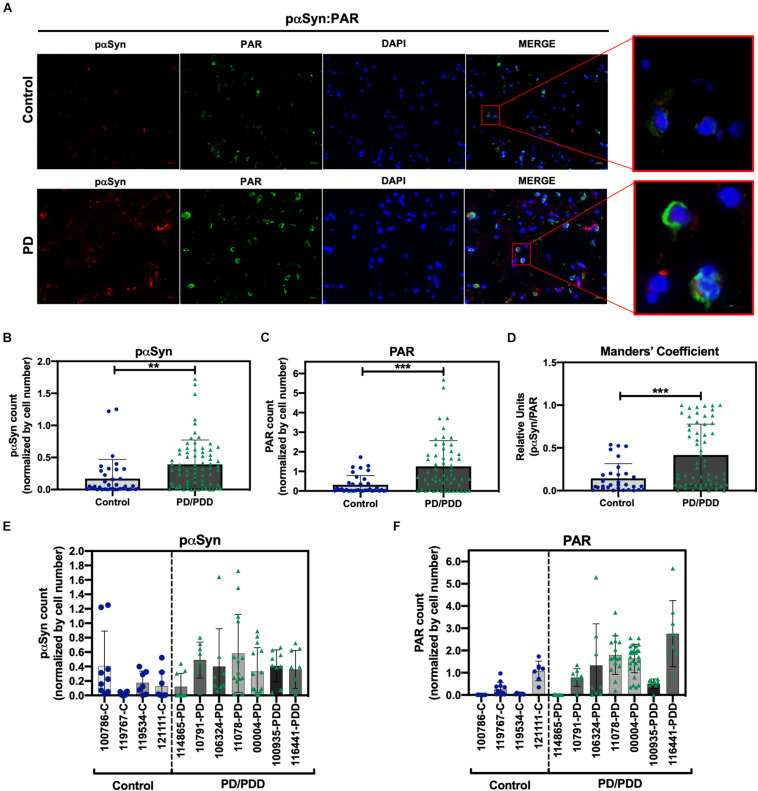
Increased S129 pαSyn and PAR levels in PD/PDD patient samples. **(A)** Representative IF immunostain of pαSyn (red), PAR (green), and DAPI (blue) in non-PD control (***top panel***, tissue ID 121111, middle frontal gyrus) and PD/PDD (***bottom panel***, tissue ID 116441, middle frontal gyrus) patient samples. Merge channel regions of interest (ROI) show colocalization between pαSyn and PAR staining. Scale bar 20 μm. Images were captured using Zeiss Axio Widefield (20×/0.8) microscope. **(B)** Quantification of pαSyn levels, normalized by DAPI count, in control vs. PD/PDD patients. **(C)** Quantification of PAR levels, normalized by DAPI count, in control vs. PD/PDD patients. **(D)** Quantification of pαSyn/PAR overlap in control vs. PD/PDD patients using Manders’ overlap coefficient. Graphical symbols represent *fields-of-view* containing 100–150 cells each. Immunostain quantification of **(E)** pαSyn and **(F)** PAR expression for all human PD/PDD and non-PD post mortem brain samples used in this study. For all experiments, bars represent means ± SD. Student’s two-tailed *t*-test [*n* = 4 (control) and 7 (PD/PDD) patient samples per group]. **P < 0.0028, ***P < 0.0001. Graphical symbols represent *fields-of-view* containing 100–150 cells each.

**FIGURE 4:**
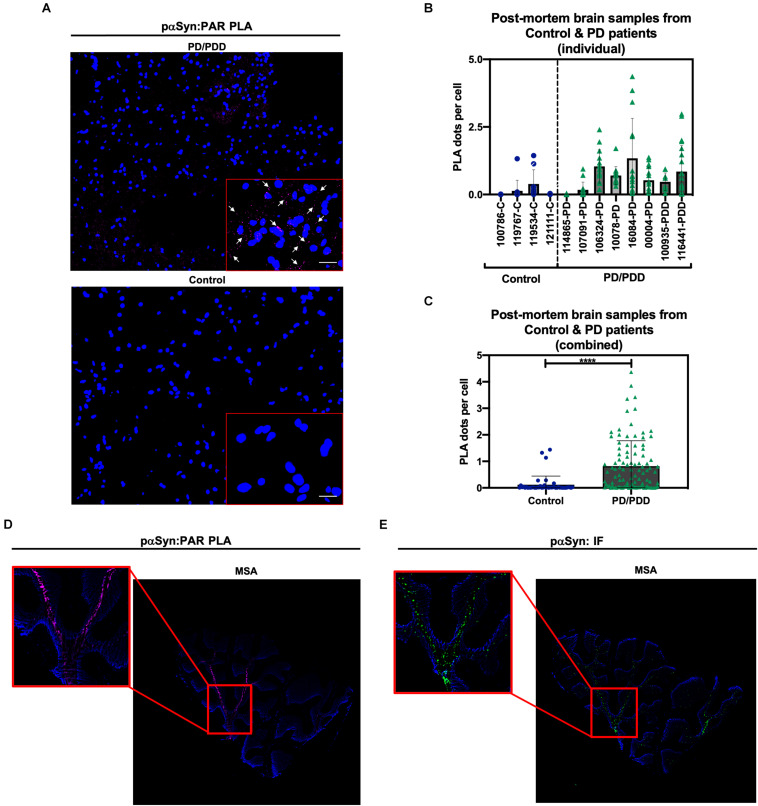
Poly (ADP-ribose) predominantly interacts with pαSyn in PD/PDD post mortem patient samples. **(A)** Representative PLA and DAPI images in non-PD control (***top panel***, tissue ID 121111, middle frontal gyrus) and PD/PDD patient samples (***bottom panel***, tissue ID 116441, middle frontal gyrus). ROI overlaid on merge channel. ROI scale bar 10 μm. Images were captured using Zeiss Axio Widefield (20×/0.8) microscope. Representative white arrows showing PLA positive signal. **(B)** Quantification of PLA dot count per cell in individual non-PD control vs. PD/PDD patient samples. Bars represent means ± SD. **(C)** Combined PLA analysis for all control and PD/PDD tissue samples. Bars represent means ± SD. Student’s two-tailed *t*-test [*n* = 4 (control) and 8 (PD/PDD) patient samples per group]. ****P < 0.0001. For **(B,C)** each graphical symbol represents the average number of PLA dots normalized to cell count (DAPI) for each *field-of-view*. 10–15 fields were captured for each patient sample. **(D)** PLA (pink) and DAPI (blue) signal from cerebellum sections derived from an MSA patient. **(E)** Standard immunostain of adjacent cerebellum sections from an MSA patient showing pαSyn (green) and DAPI (blue) channel images. Red boxes indicate PLA signal **(D)** and matching pαSyn IF **(E)** in adjacent MSA tissue sections. Images were captured using Zeiss Axio Widefield (20×/0.8) microscope.

To determine the clinical relevance of PAR–pαSyn interactions in PD, we performed immunoassays (Figures 3A–F) and PLA (Figures 4A–C) on human post mortem striatum, midfrontal gyrus, and hippocampus brain regions derived from PD and PDD (Parkinson’s Disease Dementia) patients, as well as, non-PD controls. Results from these studies revealed heterogeneity in pαSyn (Figure 3E) and PAR (Figure 3F) immunostaining for all patient samples, however, PD/PDD patient samples had overall increased pαSyn and PAR expression when compared to control (Figures 3A,B). We also observed higher signal overlap (i.e., colocalization) between PAR and pαSyn staining in the PD/PDD patient samples, as determined by Manders’ overlap coefficient (Figure 3D). Cumulatively, the PD/PDD patient group had higher pαSyn (Figure 3B), PAR (Figure 3C), and PLA (Figures 4A–C) signal. Interestingly, we found that patients with pathological αSyn scores classified as severe (3+) (McKeith et al., 2017) had higher PAR (Figure 3F) and PLA (Figure 4B) signal output (*patient information can be found in Supplementary Table 3*). Thus, suggesting that PLA signal may be associated with disease progression and severity.

To supplement our PD/PDD PLA results, we also performed PAR–pαSyn PLA (Figure 4D) and pαSyn immunostains (Figure 4E) on cerebellum tissue samples from patients diagnosed with multiple system atrophy (MSA) – another main type of α-synucleinopathy – along with healthy region-matched controls (Supplementary Figures 3A,B). From this, we observed PLA staining patterns that closely matched pαSyn pathology in MSA (Figures 4D,E). As a result, we were able to validate PLA signal in two different α-synucleinopathies. From our studies, we found that including technical controls (i.e., imaging adjacent tissue sections in the absence of primary antibodies), along with validation of signal in more than one disease type, led to reliable results from our PLA studies.

In summary, although there was heterogeneous expression of pαSyn and PAR in the patient tissue samples, we found there to be a statistically significant increase in PLA signal in the PD/PDD patient group relative to age-matched controls. *The data obtained from these experiments, to our knowledge, is the first direct evidence showing pathologically relevant PAR–pαSyn associations on human post mortem brain tissue samples from PD/PDD and MSA patient groups*. Further studies are warranted in order to better understand the role of PAR-bound pαSyn in the disease progression of PD/PDD and other synucleinopathies. Such a finding could have wide ranging implications for the development of disease modifying therapies (such as PARP-1 inhibitors) for patients harboring familial PD genetic variants (i.e., A30P, E46K, and A53T).

### PAR Binds αSyn via Electrostatic Interactions Involving Lysine Residues

**FIGURE 5:**
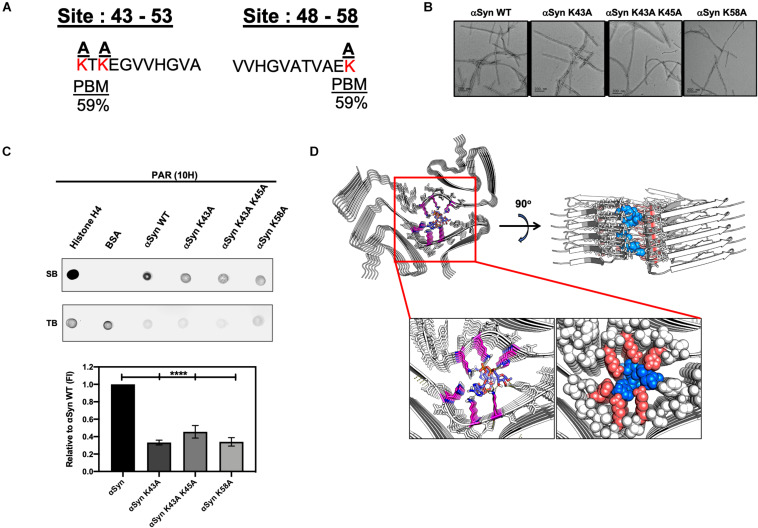
Poly (ADP-ribose) interacts with αSyn via electrostatic forces in the N-terminal region of the protein. **(A)** Alignment of the full αSyn sequence with PBMs yielded a 59% PAR-binding probability at amino acid residues 43–54 and 48–58 on αSyn. Lysine amino acid residues (red) at the two sites were substituted to neutral alanine residues. **(B)** Three mutants of αSyn were generated with compromised PAR-binding sites. Two of the αSyn mutants had a point mutation at amino acid residues K43 and K58, respectively, while the third mutant had two point mutations at positions K43 and K45. All mutants were fully fibrillated within 72 h. Scale bar 200 nm. **(C)** PAR immunodot blot (***top panel***), whereby WT and mutant αSyn fibrils were spotted onto a membrane, along with, histone H4 (positive control), and BSA (negative control) and incubated with PAR polymer to assess PAR binding. Semi-quantitative analysis (***bottom panel***) of WT and mutant αSyn fibril signal intensity normalized to WT αSyn signal. One-way ANOVA (*n* = 3). ****P < 0.0001. **(D)** Cryo-EM structure of MSA Type I αSyn fibril interacting with the PAR-dimer complex with a low free binding energy of –15.6 kcal/mol.

A previous study (Kam et al., 2018) showed that PAR binds αSyn via non-covalent interactions on the N-terminal region of αSyn – thus, suggesting that the interactions between PAR and αSyn are electrostatic in nature. In order to identify the amino acid residues involved in αSyn-PAR binding, a protein alignment tool (NPS@PATTINPROT search) was used to align the native αSyn protein sequence to two published PAR binding motifs (PBM) (Pleschke et al., 2000; Gagné et al., 2008). We identified two sites on αSyn as potential PAR-binding sites (Figure 5A): a site between amino acids residues 43–54 and another site between amino acid residues 48–58.

To better characterize αSyn-PAR interactions, we substituted positively charged lysine residues at the two PAR-binding sites predicted by the NPS@PATTINPROT tool (Figure 5A). Using site-directed mutagenesis, we generated three mutants of αSyn with compromised PAR-binding sites by replacing lysine residues with neutral alanine residues (Figure 5A). Two of the αSyn mutants had a single point mutation at amino acid residues K43 and K58, respectively, while the third mutant had two point mutations at positions K43 and K45 (Figures 5A,B). To test if PAR binding was affected in the different αSyn mutant fibrils, we performed PAR-binding immunodot blot (Figure 5C). WT and mutant αSyn fibrils were spotted onto a membrane along with a PAR-binding protein, histone H4 (positive control) and BSA (negative control). Incubation with PAR polymer, followed by immunoblotting with a PAR-specific antibody (10H), revealed that both histone H4 and αSyn WT fibril bound to PAR. Interestingly, we observed a *decrease in PAR binding for all three mutants* when compared to αSyn WT fibril (Figure 5C).

To assess if the decrease in PAR binding on the alanine mutant fibrils was a direct result of substituting positively-charged lysine residues – and not just due to the introduction of a point mutation, we performed an additional PAR-binding immunodot blot with αSyn A53T fibrils. From this, we observed that PAR binds the αSyn A53T mutant with similar signal intensity compared to αSyn WT fibril (Supplementary Figure 3C). Based on these results, we confirm that PAR binding to αSyn is primarily mediated by *electrostatic interactions at positions 43–58 of the N-terminal region*. Our data also indicates that PAR binds to a known familial point mutation of αSyn (A53T) (Supplementary Figure 3C). The latter has direct relevance in patients who harbor the A53T variant of αSyn – as this variant has been shown to be aggregation prone and has been directly linked to autosomal dominant early onset PD.

### PAR, MSA, and Beyond

The cryo-EM structure of Sarkosyl-insoluble αSyn filaments isolated from five MSA cases was recently reported (Schweighauser et al., 2020). The structure revealed two different filament types, type I and type II. Both filament types consist of two different protofilaments having an extended N-terminus and compact C-terminal body. In addition, the interface between the two different protofilaments consist of a non-proteinaceous density in the region of K43 and K45 in one protofilament, and K58 of the other protofilament. Since replacement of these lysine residues with an alanine residue diminished PAR binding (Figure 5C), and since PAR and pαSyn interact in both PD/PDD (Figures 4A–C) and MSA brain tissue (Figure 4D), we conducted a series of computational chemistry studies (Figure 5D) to evaluate the interaction of a PAR-dimer (Supplementary Figure 3D) with the cryo-EM structure of αSyn, specifically the type I filament (Schweighauser et al., 2020). From this, we found a very strong fit of the PAR-dimer in the space occupied by this non-proteinaceous density – with strong ionic interactions between these lysine residues and the diphosphate moiety of the PAR-dimer and hydrogen bond interactions between tyrosine-39 and histidine-50 with the adenine group and ribose groups (Figure 5D). Based on these results, it is conceivable that the non-proteinaceous density in the cryo-EM structure of αSyn filaments reported in MSA *may be PAR*; however, additional research is warranted to confirm this hypothesis.

## Discussion

Poly (ADP-ribose) is a highly branched polymer that has been best characterized as a recruiter of DNA repair factors during single-strand DNA break repair. However, in recent years the role of PAR outside of the nucleus has become clearer, and the role of this polymer in neurodegeneration stands as a promising avenue for better understanding the molecular basis of neurotoxicity leading to neurodegeneration. Specifically, the interaction between PAR and αSyn may represent a critical step in the formation of Lewy bodies and Lewy neurites. Our observation that significant interactions between PAR–pαSyn are present in post mortem PD/PDD and MSA patient samples suggests that this interaction may be prevalent in disease. Understanding this interaction and relating it to disease progression may have wide implications in treating the synucleinopathies since non-toxic PARP inhibitors could represent an important disease-modifying therapy (Berger et al., 2018).

Another novel finding in our present work is the identification of the amino acid residues that could be responsible for the binding of PAR to αSyn. Our studies confirm that PAR and αSyn interact via electrostatic forces involving positively charged lysine residues on αSyn. Our computational chemistry studies also suggest that the non-proteinaceous density in αSyn fibrils isolated from MSA brain may be PAR; additional studies are clearly needed to confirm this hypothesis.

In summary, our results confirm previously reported findings, suggesting that PAR is involved in the formation of disease-associated αSyn aggregates in PD. Furthermore, our studies are significant since they represent the first demonstration of the presence of PAR–pαSyn interactions in post mortem samples of PD, PDD, and MSA brain. Our data also suggest that the interaction of PAR with αSyn occurs via the electrostatic interactions of negatively charged PAR with positively charged lysine residues toward the N-terminus of the protein. The computational chemistry studies described here also suggest that the non-proteinaceous density found in αSyn isolated from MSA brain may be PAR and suggest further studies aimed at confirming this hypothesis.

### Study Design

The primary objectives of this study were to investigate PAR binding to pαSyn in cell and murine models of αSyn aggregation, as well as human post mortem brain samples. In addition, we also aimed to identify the amino acid residues involved in αSyn–PAR binding. These controlled laboratory experiments involved the use of immunostaining, PLA, molecular biology, and computational chemistry techniques.

Immunostaining and PLA results were analyzed using CellProfiler 3.0 (McQuin et al., 2018) software, whereby, specialized pipelines were implemented to identify and count PAR and pαSyn staining (Cell/particle counting pipeline) or PLA dot signal (modified Speckle Counting pipeline; whereby PLA dots were identified within cells’ cytoplasm). For animal studies, sample size for each age group was *n* = 3. Age groups were determined via PAR immunostaining, whereby, mice that were 12 months of age and older displayed higher PAR intensity. The oldest mice in our study were between 17 and 18 months of age, therefore, we defined 17 months as the end point for our murine data collection. Mice that were older than 12 months were included in the “aged” group, whereas mice that were younger than 12 months were included in the “young” group. Similarly, we used littermate controls for the “aged” group to account for age-related effects. Information on the strain, sex, and age of the mice used in this study can be found in Supplementary Table 2. For studies on post mortem tissue samples, PD/PDD cases were characterized by PD type pathology (Supplementary Table 3).

## Materials and Methods

### PAR Polymer

Purified PAR polymer chains (commercially obtained from TREVIGEN) were synthesized from PARP-1 in the presence of NAD^+^, cleaved and subsequently purified. PAR chain lengths ranged in size from 2 to 300 ADP-ribose subunits, with a final concentration of 10 μM.

### Cell Culture

SH-SY5Y-αSyn cells were transfected using Lipofectamine 2000 (Invitrogen) with pcDNA3.1 expression vector following manufacturer’s protocol. The expression vector contained the full-length human wild type αSyn cDNAs, cloned in the polylinker region at the KpnI and ApaI sites. Stable transfected cell lines were selected and maintained in complete medium containing 300 μg/ml G418 (Invitrogen) (Mazzulli et al., 2006). The cells were maintained in DMEM/F12 media with GlutaMAX supplement (Thermo Fisher Scientific, Cat#10565018), 10% heat-inactivated fetal calf serum (FBS), 100 units/ml penicillin and 100 μg/ml streptomycin (Pen-Strep), in a humid atmosphere of 5% CO_2_ and 95% O_2_ at 37°C.

### *BioPORTER* Experiments

SH-SY5Y-αSyn cells were seeded at concentrations of 16,000 cells/well in Nunc^®^ Lab-Tek Chamber Slide^TM^ system (8 wells, 0.8 cm^2^/well) (Millipore, C7182-1PAK) for fluorescent microscopy experiments (IF and PLA) 24 h before incubation with either PAR + *BioPORTER*, ADP-HDP + *BioPORTER* or *BioPORTER* alone (vehicle control). *BioPORTER* Protein Delivery Reagent “QuikEase Kit” (Genlantis, Cat#BP502424) was prepared according to the manufacturer’s protocol, briefly described as follows. Either PAR or ADP-HDP were diluted in 100 μL PBS to a final concentration of 50 nM, the diluted solution was then added to a QuikEase Single-Use Tube containing the dried BioPORTER reagent, mixed by pipetting 3–5 times, incubated at room temperature (RT) for 5 min and gently vortexed (post-incubation) for 3–5 s. Opti-MEM I Reduced Serum Medium (Life Technologies Inc., Cat#31985062) was used to bring the final volume in each QuikEase Single-Use Tube to 500 μL. The cells were washed once with Reduced Serum Medium 1 h before *BioPORTER* delivery, then replenished with 200 μL of Opti-MEM I (Thermo Fisher Scientific, Cat#31985062). *BioPORTER* medium mix was added at a 1:1 volume ratio in the cells grown in chamber slides. The cells were subsequently incubated for 4 h at 37°C. After 4 h, one volume of 20% serum-containing medium was added directly to the chamber slides, 24 h post-*BioPORTER* delivery, the medium was aspirated from the chamber slides and replenished with complete growth medium (DMEM/F12 media with GlutaMAX supplement). 48 h after *BioPORTER* delivery, the cells were washed 2× with PBS and processed for downstream experiments.

### Animals

M83-SNCA^∗^A53T mice expressing human A53T variant αSyn were obtained from The Jackson Laboratory, Bar Harbor, ME (JAX stock #004479). All mice were on B6;C3H genetic background. Animals were housed under controlled temperature and lighting conditions and had free access to food and water. All animal procedures were approved by IACUC and were in accordance with the National Institutes of Health Guide for the Care and Use of Laboratory Animals.

### Human Post Mortem Brain αSyn Pathology Analysis

Human brain samples were obtained from University of Pennsylvania’s Center for Neurodegenerative Disease Research Brain Bank and were evaluated with standardized histopathological methods as described (Toledo et al., 2014; Brettschneider et al., 2015; Robinson et al., 2018).

### Immunofluorescence (IF) Staining

Forty-eight hours post-*BioPORTER* delivery, SH-SY5Y-αSyn cells (seeded on chamber slides at 16,000 cells per well) were fixed on ice with 4% paraformaldehyde for 8 min. The cells were then washed 3× with PBS and permeabilized with 0.1% Triton X-100 for 10 min at RT. After permeabilization, the cells were washed 3× with PBS-T (PBS with 0.1% Tween-20) at RT. After the third wash, 200 μL of 10% goat serum (Thermo Fisher, Cat#50062Z) was added to each well for 1 h at 37°C to block non-specific immuno binding. After blocking, the cells were sequentially incubated with primary antibodies (Supplementary Table 1) targeting PAR (10H) and pαSyn (ps129) overnight at 4°C. Following primary antibody incubation, the cells were washed 3X with PBS-T. After the third wash, the cells were then sequentially incubated with secondary antibodies (Supplementary Table 1) for 1 h at 37°C, washed 3X with PBS-T, and stained with DAPI. Coverslips were placed on each slide and the slides were allowed to dry overnight at 4°C. Images were captured using Zeiss LSM 710 confocal (40x/1.4 Oil) and Zeiss Axio Widefield (20x/0.8) microscopes.

Human post mortem tissue sections were treated with TrueBlack (TrueBlack Lipofuscin Autofluorescence Quencher) according to manufacturer’s protocol, in order to eliminate lipofuscin autofluorescence before immunostaining.

Following the blocking step with 10% goat serum, murine tissue sections underwent an additional blocking step with anti-mouse IgG (Supplementary Table 1) for 1 h at 37°C in order to reduce non-specific signal from secondary antibodies directed against PAR antibody (10H), which is a mouse monoclonal primary antibody.

### Proximity Ligation Assay (PLA)

Forty-eight hours post-*BioPORTER* delivery, chamber slides cells (SH-SY5Y-αSyn cells) were processed with regards to fixation and permeabilization using the IF protocol described in the previous section. *In situ* PLA was performed according to the manufacturer’s protocol, briefly described as follows. Following permeabilization, cells were blocked using Duolink^®^ Blocking Solution for 1 h at 37°C. PAR primary antibody (Supplementary Table 1) was diluted in Duolink^®^ Antibody Diluent, added to the cells and incubated overnight at 4°C. Following overnight incubation with PAR primary antibody, the cells were washed 2× with Duolink^®^ Wash Buffer A, then incubated with Duolink^®^ PLA Probe (goat anti-mouse *MINUS*) for 1 h at 37°C. After incubation with PLA Probe *MINUS*, the cells were washed 2× with Wash Buffer A, blocked with Duolink^®^ Blocking Solution for 1 h at 37°C and incubated overnight at 4°C with primary antibody targeting pαSyn (Supplementary Table 1). Following overnight incubation, the cells were washed 2X with Wash Buffer A and incubated with Duolink^®^ (PLA Probe goat anti-rabbit *PLUS*) for 1 h at 37°C. Following the sequential addition of primary antibodies and corresponding PLA Probes, the cells were processed with respect to ligation (Duolink^®^ Ligation buffer and Ligase), amplification (Duolink^®^ Amplification buffer and Polymerase) and imaging using Zeiss Axio Widefield (20×/0.8) microscope. Human post mortem tissue sections were treated with TrueBlack (TrueBlack^®^ Lipofuscin Autofluorescence Quencher) according to manufacturer’s protocol, in order to eliminate lipofuscin autofluorescence before PLA. Murine tissue sections underwent an additional blocking step with anti-mouse IgG (Supplementary Table 1) for 1 h at 37°C to reduce non-specific signal from goat anti-mouse *MINUS*.

### αSyn Protein Expression and Purification

Protein expression and purification was done following previously published protocol (Lengyel-Zhand et al., 2020). Briefly, the plasmid encoding the human αSyn sequence was transformed into *Escherichia coli* BL21(DE3) and the cells were grown on agar/LB plates with ampicillin (100 μg/mL) overnight at 37°C. The next day a single colony was inoculated into 100 mL Luria–Bertani (LB) containing ampicillin (100 μg/mL). The culture was incubated at 37°C overnight with shaking at ∼200 rpm. The following day, 10 mL of the overnight culture was diluted with 1 L of LB media supplemented with ampicillin and this culture was incubated at 37°C until OD_600_ reached 0.6 – 0.7. Protein expression was induced by addition of isopropyl-β-D-thiogalactoside (IPTG) to a final concentration of 1 mM and continued to grow at 18°C overnight. After induction, cells were harvested by centrifugation at 4°C (20 min, 4,000 *g*). The typical yield of wet-cell paste was 2 g/L. Cells were suspended in a lysis buffer (5 mL for 1 g of cell paste) containing 25 mM Tris, 20 mM imidazole, 50 mM NaCl (pH 8) with a protease inhibitor (phenylmethylsulfonyl fluoride, 0.5 mM final concentration and protease inhibitor cocktail from Cell Signaling Technology). Cells were lysed by sonication on ice for 10 min (20 s on, 20 s off). The crude cell lysate was then centrifuged at 20,000 *g* for 30 min, and the supernatant was mixed with Ni-NTA resin (Clontech, 3 mL) and kept on a rocker at RT for 30 min. The resin was then washed with 100 mL wash buffer (25 mM Tris, 20 mM imidazole, 50 mM NaCl, pH 8). The protein was eluted with a buffer containing 25 mM Tris, 300 mM imidazole, 50 mM NaCl (pH 8). Fractions containing the protein were identified by UV-Vis spectroscopy, combined and was treated with β-mercaptoethanol (200 mM final concentration) overnight at RT to cleave the C-terminal intein. The next day, the protein was concentrated to 3 mL and dialyzed against buffer containing 25 mM Tris, 50 mM NaCl, pH 8. After dialysis, the protein mixture was loaded onto Ni-NTA column and the pure αSyn protein was collected in the flow through fractions. The combined protein fractions were concentrated and dialyzed against buffer containing 50 mM Tris, 150 mM NaCl, pH 7.5. The purity of the protein was confirmed by SDS-PAGE. Protein concentration was determined by measuring the absorbance at 280 nm and using the calculated (ExPASy) extinction coefficient of 5,960 M^–1^cm^–1^.

### Site-Directed Mutagenesis

αSyn mutations were generated by performing site directed mutagenesis using the following primers:

**αSyn K43A**
Forward: 5′- TCCGCAACCAAGGAGGGA -3′
Reverse: 5′- TCCCTCCTTGGTTGCGGA - 3′
**αSyn K43A K45A**
Forward: 5′- GGCTCCGCAACCGCGGAGGGAGTG - 3′
Reverse: 5′- CACTCCCTCCGCGGTTGCGGAGCC - 3′
**αSyn K58A**
Forward: 5′-GTGGCTGAGGCGACCAAA - 3′
Reverse: 5′-TTTGGTCGCCTCAGCCAC - 3′

All plasmids and inserts were sequenced and confirmed to be free of any errors.

### PAR Binding Motifs (PBM)

*hxbxhhbbhhb* (h are hydrophobic residues, b are basic residues, and x is any amino acid residue) (Pleschke et al., 2000). [HKR]-X-X-[AIQVY]-[KR]-[KR]- [AILV]-[FILPV] (Pleschke et al., 2000; Gagné et al., 2008).

### Preparation of αSyn Fibrils

Purified αSyn monomer (100 μM) was incubated in buffer containing 50 mM Tris (pH 7.5), 150 mM NaCl and 0.05% NaN_3_ for 72 h at 37°C with shaking at 1,000 rpm in a Fisher Scientific Mixer.

### Transmission Electron Microscopy (TEM)

The 100 μM fibril stock solution was diluted 4× with water and samples (5 μL) were spotted onto glow-discharged formvar/carbon-coated, 200-mesh copper grids (Ted Pella). After 1 min, grids were washed briefly with water and stained with one 10 μL drop of 2% w/v uranyl acetate for 1 min. The excess stain was removed by filter paper and the grids were dried under air. Samples were imaged with a Tecnai FEI T12 electron microscope at an acceleration voltage of 120 kV. Images were recorded on a Gatan OneView 4K Cmos camera.

### PAR Immunodot Blot

PAR-binding motif (PBM) were identified by aligning the PBM consensus to αSyn using the PATTINPROT search engine (NPS@PATTINPROT). For immunodot analysis, either 1 mg fibrils, Histone H4 (positive control), or bovine serum albumin (negative control) were blotted onto a 0.2 μm nitrocellulose membrane (Bio-Rad). Membranes were left to dry for 60 min, then incubated in DPBS supplemented with 0.05% Tween-20 (PBS-T) for 10 min. The membrane was then incubated with 50 nM PAR polymer in PBS-T for 2 h with rocking at RT. The membrane was washed 5× with PBS-T (5 min each) and blocked with PBSMT (5% milk in PBS-T) for 2 h at RT. After the blocking step, the membrane was incubated in primary antibody (Supplementary Table 1) in PBS-T at 4°C overnight. After 5 washes in PBSMT (5 min each), the membrane was incubated with secondary antibody (Supplementary Table 1) in PBSMT for 1 h at RT. The membrane was washed 3× in PBSMT, 2× in PBS-T, and 2× in DPBS (5 min each). The membrane was then imaged using Li-COR ODYSSEY CLx scanner. Spot intensities were measured using Image Studio software. Revert 700 protein stain was used for total protein staining measurement. Blotted membranes were incubated with protein stain for 5 min, rinsed with Revert 700 wash buffer, and imaged using Li-COR ODYSSEY CLx scanner.

### Molecular Docking

The PAR-dimer structure used in our studies was based on Lambrecht et al. (2015) and drawn on ChemDraw Profession 15.1 (PerkinElmer Informatics, Inc.). It was then imported to Chem3D Ultra 15.1 (PerkinElmer Informatics, Inc.) to minimize the PAR-dimer by MMFF94 force field for preparation of molecular docking. Molecular docking studies were performed via AutoDock 4.2 (Morris et al., 2009) plugin on PyMOL^1^. Cryo-EM structure of MSA Type I αSyn fibril (PDB ID 6XYO, Resolution 2.6 Å) was obtained from RCSB Protein Data Bank^2^. Polar hydrogens were added to the fibril structure. Non-polar hydrogens were removed from the PAR-dimer. A grid box with a dimension of 30 × 30 × 30 Å^3^ was applied to the MSA Type I αSyn fibril structure covering the non-proteinaceous density pocket at the protofilament interface. The Lamarckian Genetic Algorithm with a maximum of 2,500,000 energy evaluations was used to calculate 100 αSyn fibril-PAR binding poses. The αSyn fibril-PAR complex with the most contacts and low free binding energy was reported.

### Quantification and Statistical Analysis

All measurements were taken from distinct samples. Data points in each graph are mean (±SD); where “*n*” indicates the number of biological replicates for each experiment. *T*-tests, one-way ANOVA, and two-way ANOVA followed by Tukey’s *post hoc* test were performed and are described in each figure legend. Statistical significance was set at *P* < 0.05. All statistical analyses were carried out using Graphpad prism 8 software.

## Data Availability Statement

The original contributions generated for this study are included in the article/Supplementary Material, further inquiries can be directed to the corresponding author.

## Ethics Statement

The animal study was reviewed and approved by IACUC and was in accordance with the National Institutes of Health Guide for the Care and Use of Laboratory Animals.

## Author Contributions

LP performed all the cell-based studies and *ex vivo* animal and patient experiments, along with the computational protein alignment. ZL-Z helped to produce all the purified proteins and fibrils used in this project and performed TEM, ThT, and PAR immunodot blot analysis. LP and ZL-Z designed the primers for mutagenesis. JL maintained cell cultures and aided in the experimental set-up for PLA. C-JH performed molecular docking studies. MS maintained αSyn protein expression and purification. KE provided assistance with PLA. KL provided support with animal model and experimental design. VL and JT provided support with experimental design and characterization of human post mortem brain tissue from PD/PDD and non-PD patients. All authors contributed to the article and approved the submitted version.

## Conflict of Interest

The authors declare that the research was conducted in the absence of any commercial or financial relationships that could be construed as a potential conflict of interest.

## Footnotes

1 pymol.org

2 www.rcsb.org

## Supplementary Material

Supplementary Figure 1 Poly (ADP-ribose) (PAR) ELISA. **(a)** PAR ELISA on SH-SY5Y-αSyn cells treated with 50 nM PAR/*BioPORTER* at 4 and 48 h. ^∗^*P* < 0.05. Bars represent means ± SD. Student two-tailed *t*-test (*n* = 3). **(b)** PAR ELISA on SH-SY5Y-αSyn cells treated with ADP-HDP for 4, 24, and 48 h. Bars represent means ± SD. One-way ANOVA followed by Tukey’s *post hoc* test. * *P* < 0.05.

Supplementary Figure 2 Characterization of PAR and pαSyn expression in murine brain sections. Representative images of endogenous PAR **(a)** and pαSyn **(b)** levels in sagittal brain sections from B6C3F1/J (top panel) and M83 Tg (bottom panel) mice at three different age groups (8, 12, and 17 months). **(c)** PLA on PAR-pαSyn (left) and PAR-PAN-αSyn (right) sagittal brain sections from M83 Tg mice at 18 months of age. All sections were 10 μm thick and all images were captured using a Li-COR ODYSSEY CLx scanner.

Supplementary Figure 3 Cerebellum PLA and A53T dot blot. **(a)** PLA and DAPI signal from cerebellum sections derived from a control “non-MSA” patient. **(b)** Standard immunostain of adjacent cerebellum sections from a control patient showing pαSyn and DAPI signal. Images were captured using Zeiss Axio Widefield (20×/0.8) microscope. **(c)** Representative PAR immunodot blot to assess PAR-αSyn A53T binding. Signal intensity was normalized to αSyn WT signal. Histone H4 and BSA were used as positive and negative controls for PAR binding, respectively. **(d)** Chemical structure of PAR-dimer used in molecular docking studies.

Supplementary Table 1 Antibody information.

Supplementary Table 2 Animal information.

Supplementary Table 3 Patient information.

Supplementary Table 4 Microscopy settings.
