# Supplementary figures and images for: Poly (ADP-ribose) Interacts With Phosphorylated α-Synuclein in Post Mortem PD Samples
Source: Front Aging Neurosci. 2021 Jun 18;13:704041. doi: 10.3389/fnagi.2021.704041 (PMC8249773; doi:10.3389/fnagi.2021.704041)

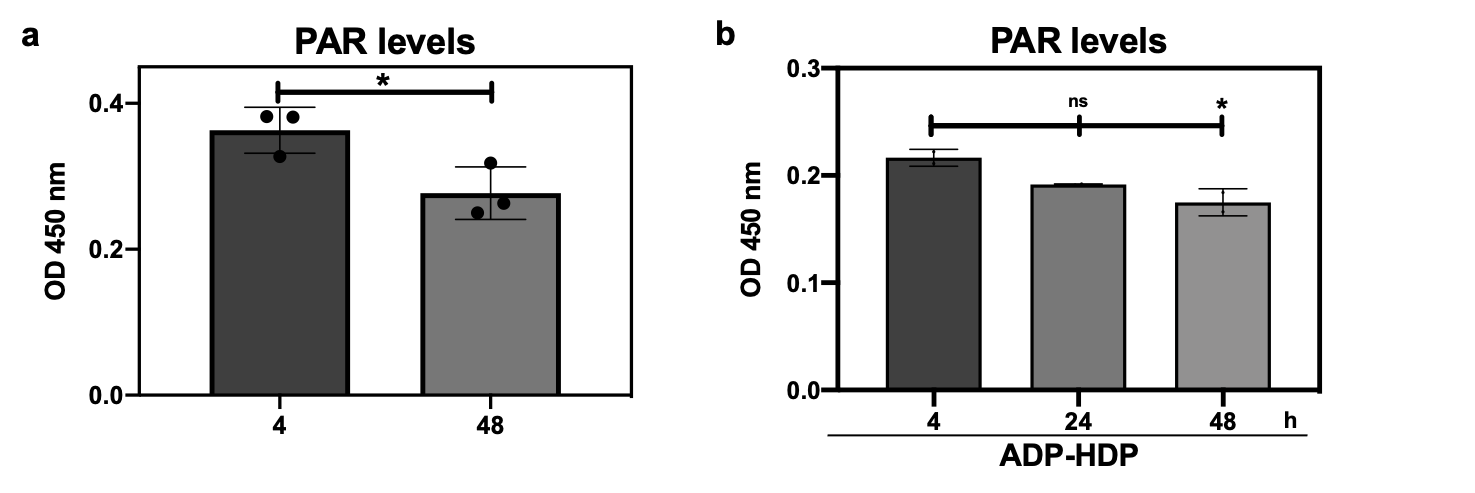

Supplement: Supplementary Figure 1 — Poly (ADP-ribose) (PAR) ELISA. (a) PAR ELISA on SH-SY5Y-αSyn cells treated with 50 nM PAR/BioPORTER at 4 and 48 h. ∗P < 0.05. Bars represent means ± SD. Student two-tailed t-test (n = 3). (b) PAR ELISA on SH-SY5Y-αSyn cells treated with ADP-HDP for 4, 24, and 48 h. Bars represent means ± SD. One-way ANOVA followed by Tukey’s post hoc test. * P < 0.05. [file Image_1.TIFF]

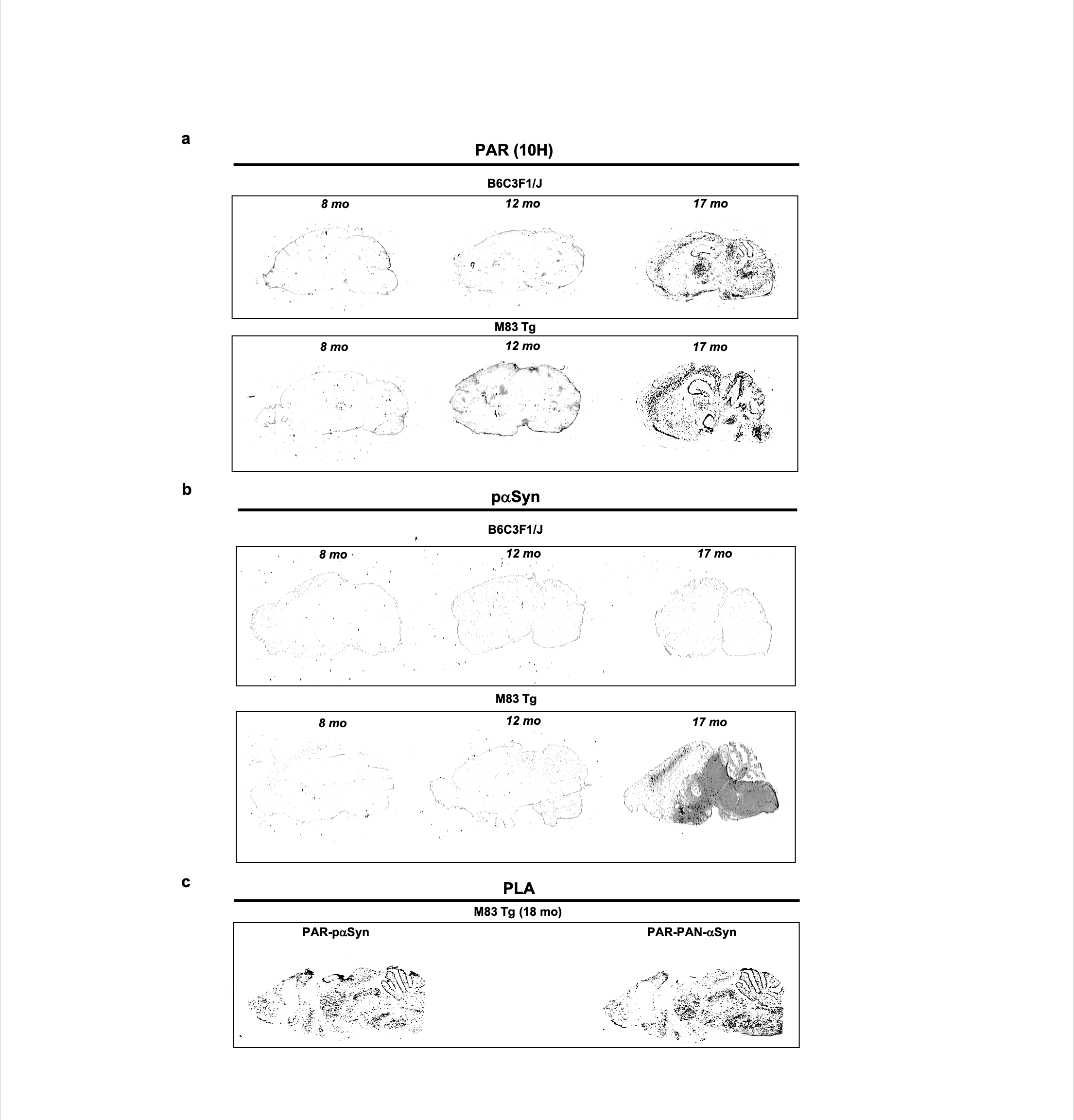

Supplement: Supplementary Figure 2 — Characterization of PAR and pαSyn expression in murine brain sections. Representative images of endogenous PAR (a) and pαSyn (b) levels in sagittal brain sections from B6C3F1/J (top panel) and M83 Tg (bottom panel) mice at three different age groups (8, 12, and 17 months). (c) PLA on PAR-pαSyn (left) and PAR-PAN-αSyn (right) sagittal brain sections from M83 Tg mice at 18 months of age. All sections were 10 μm thick and all images were captured using a Li-COR ODYSSEY CLx scanner. [file Image_2.TIFF]

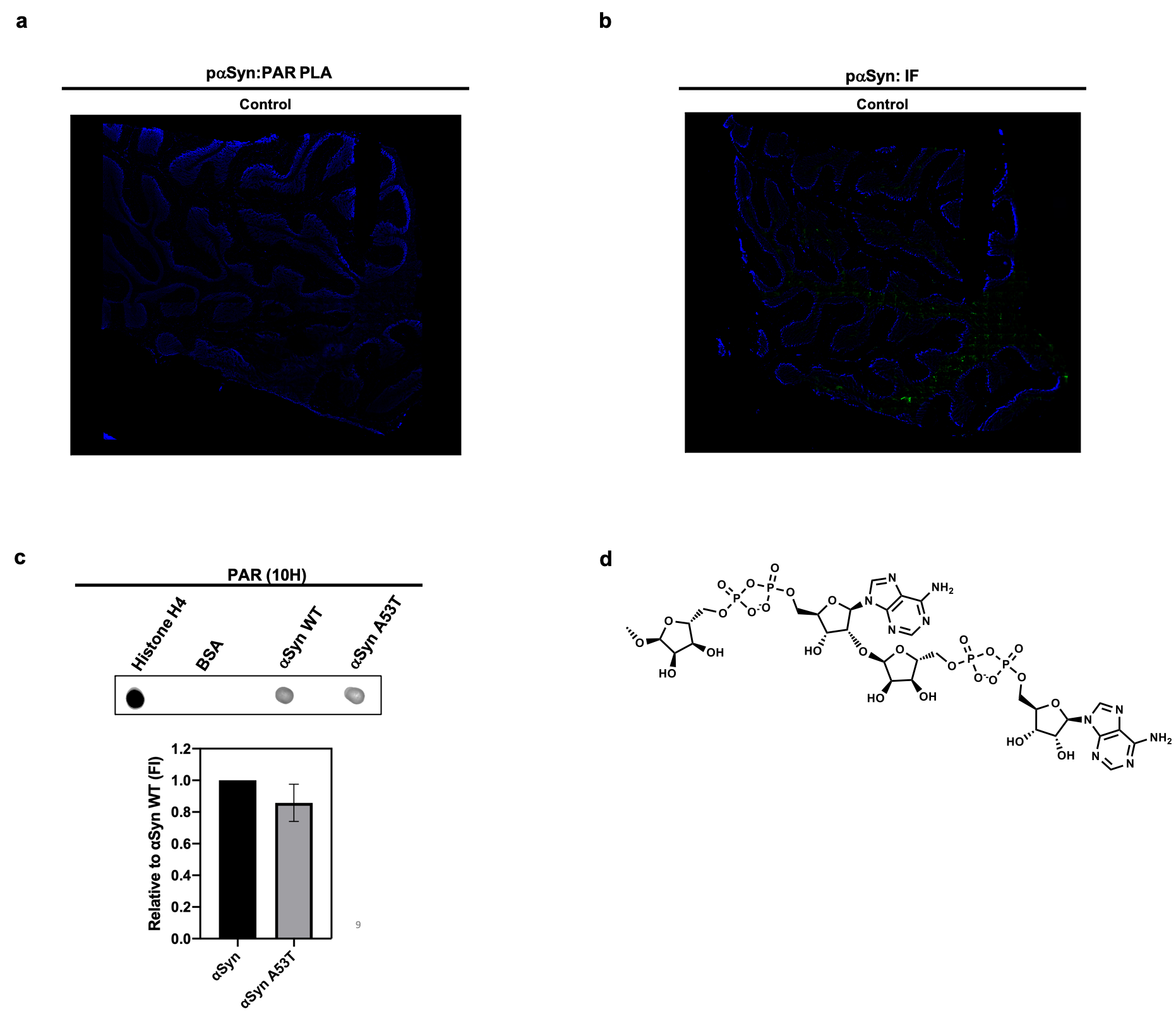

Supplement: Supplementary Figure 3 — Cerebellum PLA and A53T dot blot. (a) PLA and DAPI signal from cerebellum sections derived from a control “non-MSA” patient. (b) Standard immunostain of adjacent cerebellum sections from a control patient showing pαSyn and DAPI signal. Images were captured using Zeiss Axio Widefield (20×/0.8) microscope. (c) Representative PAR immunodot blot to assess PAR-αSyn A53T binding. Signal intensity was normalized to αSyn WT signal. Histone H4 and BSA were used as positive and negative controls for PAR binding, respectively. (d) Chemical structure of PAR-dimer used in molecular docking studies. [file Image_3.TIFF]
