# Supplementary material for: Poly (ADP-ribose) Interacts With Phosphorylated α-Synuclein in Post Mortem PD Samples
Source: Front Aging Neurosci. 2021 Jun 18;13:704041. doi: 10.3389/fnagi.2021.704041 (PMC8249773; doi:10.3389/fnagi.2021.704041)
Supplement: Supplementary Table 1 — Antibody information. [file Data_Sheet_1.docx]

**SI Tables**

**Table 1.** Antibody Information

| **Antibody** | **Antigen** | **Host Species** | **Dilution** | **Source** |
| --- | --- | --- | --- | --- |
| 81A | αSyn (Phosphorylation at Ser-129) | Mouse | 1:1,000 (IF) | Abcam |
| αSyn (PS129) | αSyn (Phosphorylation at Ser-129) | Rabbit | 1:500 (IF, PLA) | Abcam |
| PAR (10H) | PAR | Mouse | 1:500 (IF, PLA; cells), 1:1000 (IF, PLA; tissue) | Enzo |
| Goat F(ab) anti-Mouse IgG (H&l) | Mouse IgG | Mouse | 1:100 (IF & PLA; murine tissue) | Abcam |
| αSyn | αSyn (targets C-terminus, polyclonal) | Rabbit | 1:500 (PLA) | Invitrogen |
| Goat anti-Mouse IgG Alexa Fluor 488 | Mouse IgG | Mouse | 1:400 (IF) | Invitrogen |
| Goat anti-Mouse IgG Alexa Fluor 568 | Mouse IgG | Mouse | 1:400 (IF) | Invitrogen |
| Goat anti-Rabbit IgG Alexa Fluor 568 | Rabbit IgG | Rabbit | 1:400 (IF) | Invitrogen |
| Goat anti-Mouse IgG Alexa Fluor 647 | Rabbit IgG | Rabbit | 1:400 (IF) | Invitrogen |
| PLA Probe Anti-Rabbit PLUS | Rabbit IgG | Rabbit | 1:5 (PLA) | MilliporeSigma |
| PLA Probe Anti-Mouse MINUS | Mouse IgG | Mouse | 1:5 (PLA) | MilliporeSigma |

**Table 2.** Animal Information

| **Strain** | **Sex** | **Age** | **Group** |
| --- | --- | --- | --- |
| M83 SNCA*A53T | M | 6 | M83 Tg young |
| M83 SNCA*A53T | M | 7 | M83 Tg young |
| M83 SNCA*A53T | M | 8 | M83 Tg young |
| M83 SNCA*A53T | M | 12 | M83 Tg aged |
| M83 SNCA*A53T | M | 15 | M83 Tg aged |
| M83 SNCA*A53T | M | 17 | M83 Tg aged |
| ^1^B6C3F1/J | M | 12 | B6C3F1/J aged |
| ^1^B6C3F1/J | M | 12 | B6C3F1/J aged |
| ^1^B6C3F1/J | M | 17 | B6C3F1/J aged |
| ^1^non-Tg mice (littermate control) | |  |  |

**Table 3.** Patient Information

| **INDDID** | **Clinical Group** | **Age at Death** | **Sex** | **Region** | **αSyn Pathology Scores*** |
| --- | --- | --- | --- | --- | --- |
| 100786 | Control | 61 | M | Striatum | 0 |
| 119767 | Control | 83 | M | Striatum | 0 |
| 119534 | Control | 72 | F | Striatum | 0 |
| 121111 | Control | 68 | M | Middle Frontal Gyrus | 0 |
| 114865 | PD | 62 | M | Striatum | 1+ |
| 107091 | PD | 72 | F | Striatum | 2+ |
| 100935 | PDD | 80 | M | Striatum | Rare |
| 106324 | PD | N/A | N/A | Middle Frontal Gyrus | 3+ |
| 116441 | PDD | 82 | M | Middle Frontal Gyrus | 3+ |
| 10078 | PD | N/A | N/A | Hippocampus | 3+ |
| 16084 | PD | N/A | N/A | Hippocampus | 3+ |
| 00004 | PD | N/A | N/A | Hippocampus | 3+ |
| ***Semiquantitative scores by immunohistochemistry for pathological αSyn** | | |  |  |  |

**Table 4.** Microscope Settings

| ***Confocal*** |  |  |  |  |  |  |  |  |  |  |  | | | |
| --- | --- | --- | --- | --- | --- | --- | --- | --- | --- | --- | --- | --- | --- | --- |
| **Figure** | **Equipment** | **Obj. lens** | **Beam Splitter Name** | **Beam Splitter Filter** | **Acquisition software** | **Acquisition Info.** | **Bit Depth** | **Gain** | **Wavelength End/Start** | **Illumination Channel Power** | | |  |  |
| 1c | Zeiss LSM 710 | 40x/1.1 W Korr M26 | HT; MBS_  InVis | 488/561 | ZEISS, ZEN | Zeiss LSM | 8 Bit | 848 (DAPI); 566 (Alexa Fluor 488) | 417 - 484; 504 - 572 | 5 (DAPI); 1 (Alexa Fluor 488) |  | | | |
| 1e | Zeiss LSM 711 | 40x/1.1 W Korr M27 | HT; MBS_  InVis; FW1 | 488/561/633 | ZEISS, ZEN | Zeiss LSM | 8 Bit | 728 (DAPI); 598 (Alexa Fluor 488); 789 (Alexa Fluor 568) | 417 - 484; 504 - 572; 643 - 750 | 7.8 (DAPI);  0.7 (Alexa Fluor 488);  1.1 (Alexa Fluor 647) | | | | |
| ***Widefield*** |  |  |  |  |  |  |  |  |  |  | |  | | |
| **Figure** | **Equipment** | **Obj.**  **lens** | **Reflector** | **Filters** | **Acquisition software** | **Acquisition Info.** | **Bit Depth** | **Excitation** | **Emission** | **Exposure** | | **Camera** | | |
| 2d | Zeiss Axio Imager.M2 | 20x/0.8 M27 | 50 Cy 5; 49 DAPI | 625 - 655; 665 - 715; 335 - 383; 420 - 470 | ZEISS, ZEN | Carl Zeiss Image (*.czi) | 14 Bit | 625 - 655 (Cy5); 335 - 383( DAPI) | 665 - 715 (Cy 5); 420 - 470 (DAPI) | 2.8 s ms (Cy 5); 150 ms (DAPI) | | BW | | |
| 2e | Zeiss Axio Imager.M2 | 20x/0.8 M27 | 50 Cy 5; 49 DAPI | 625 - 655; 665 - 715; 335 - 383; 420 - 470 | ZEISS, ZEN | Carl Zeiss Image (*.czi) | 14 Bit | 625 - 655 (Cy5); 335 - 383( DAPI) | 665 - 715 (Cy 5); 420 - 470 (DAPI) | 600 ms (Cy 5); 190 ms (DAPI) | | BW | | |
| 2g | Zeiss Axio Imager.M2 | 20x/0.8 M27 | 50 Cy 5; 49 DAPI | 625 - 655; 665 - 715; 335 - 383; 420 - 470 | ZEISS, ZEN | Carl Zeiss Image (*.czi) | 14 Bit | 625 - 655 (Cy5); 335 - 383( DAPI) | 665 - 715 (Cy 5); 420 - 470 (DAPI) | 600 ms (Cy 5); 190 ms (DAPI) | | BW | | |
| 3a | Zeiss Axio Imager.M2 | 20x/0.8 M27 | 50 Cy 5; 38 GFP; 49 DAPI | 625 - 655; 665 - 715; 450 - 490; 500 - 550; 335 - 383; 420 - 470 | ZEISS, ZEN | Carl Zeiss Image (*.czi) | 14 Bit | 625 - 655 (Cy5); 450 - 490 (GFP); 335 - 383( DAPI) | 665 - 715 (Cy 5); 500 - 550 (GFP); 420 - 470 (DAPI) | 1.6 ms (Cy 5); 300 ms (GFP); 190 ms (DAPI) | | BW | | |
| 4a | Zeiss Axio Imager.M2 | 20x/0.8 M28 | 50 Cy 5; 49 DAPI | 625 - 655; 665 - 715; 335 - 383; 420 - 470 | ZEISS, ZEN | Carl Zeiss Image (*.czi) | 14 Bit | 625 - 655 (Cy5); 335 - 383( DAPI) | 665 - 715 (Cy 5); 420 - 470 (DAPI) | 1.6 ms (Cy 5); 150 ms (DAPI) | | BW | | |
